# Transthesia: Comparing the Prevalence of Synesthesia in Transgender and Cisgender Individuals

**DOI:** 10.1089/trgh.2018.0010

**Published:** 2018-08-01

**Authors:** Jay P. Pierce

**Affiliations:** Student, Ballard High School, Seattle, Washington.

**Keywords:** autism, gender, gender identity, survey, synesthesia, transgender

## Abstract

**Purpose:** To evaluate the prevalence of synesthesia in transgender versus cisgender individuals.

**Methods:** A 10-question, self-administered written survey, developed to assess the prevalence of synesthesia, was distributed to transgender individuals (*n*=96) attending support groups as well as to cisgender participants (*n*=103) identified among individuals accompanying transgender attendees. Demographic data and prevalence of synesthesia were analyzed using descriptive statistics. Differences between groups were analyzed using a chi-square test.

**Results:** Forty-two percent of transgender participants endorsed synesthesia compared with 16% of cisgender participants. These findings persisted when analyzed by specific gender identity (i.e., male, female, and nonbinary).

**Conclusion:** This study suggests a correlation between synesthesia and transgender identity that may indicate a common biological cause. Limitations of this study include use of a survey that has not yet been validated. Initial findings may justify further research.

## Introduction

### Synesthesia

Synesthesia is a neurological condition in which stimulation of one sensory or cognitive pathway leads to automatic, involuntary sensations in a second sensory or cognitive pathway. Synesthetes may experience colors when they hear sounds, see colors when they hear letters or words, visualize numbers as points in space or geometric shapes, “feel” sounds as a tapping on the shoulder, attach scents or tastes to human personalities, and many more.^1,2^ Once believed to be extremely rare, recent estimates suggest a prevalence of 4.4%.^3^

Synesthesia can be detected by testing the consistency of these sensations over long time intervals.^3^ The online Synesthesia Battery, which assesses consistency within a single test session, is also a valid method for assessing synesthesia.^4^ Synesthetes can be identified as early as age six.^5^

### Autism

Autism appears to have a biological component.^6,7^ Research is proceeding to develop a diagnostic test (examining brain waves, blood, or urine) for autism spectrum disorders.^8,9^

### Transgenderism

Research suggests biological factors in transgenderism.^10–12^ However, research into objective methodology for assessing transgenderism, especially in children, is still preliminary.^13^

### Correlations among synesthesia, autism, and transgenderism

Synesthesia is more common in individuals with autism.^1,14^ Individuals with autism are also more likely to be transgender or gender variant.^15–17^ No studies could be found discussing whether synesthesia and transgenderism are correlated.

Correlation of transgenderism to syndromes with publically accepted biological causes might increase societal acceptance of transgender individuals. Correlation also might assist in developing valid methods for earlier, accurate identification of transgender children, possibly improving these individuals' access to appropriate medical treatment and providing their parents with a longer period of time to access information to support their children. The aim of this study was therefore to identify whether synesthesia and transgenderism are correlated.

## Methods

### Participants and procedures

A 10-question survey was distributed to 199 transgender and cisgender attendees at 8 transgender support groups in King and Pierce Counties in Washington State during January and February 2018. Cisgender participants were generally parents and friends of transgender participants. Participant demographics are summarized in Table 1. The majority of transgender participants were age 11–30 years (65, 68%), while the majority of cisgender participants were age 31–50 years (65, 66%).

**Table 1.** **Demographic Characteristics of Survey Participants (*n*=199)**

	Transgender	Cisgender	Total
Gender identity (*n*, %)			
Male	(32, 16)	(33, 16.5)	(65, 32.5)
Female	(33, 16.5)	(66, 33)	(99, 49.5)
Nonbinary^a^	(31, 15.5)	(4, 2)	(35, 17.5)
Age (mean, range)	(26.98, 8–80)	(40.82, 9–84)	(34.08, 8–84)

^a^ “Nonbinary” indicates participants who described their gender identity as something other than “male” or “female” (Q2).

The Institutional Review Board for the Washington State Science and Engineering Fair approved the survey and the research plan, including the use of human participants. Written informed consent was obtained from all participants. Written parental consent was also obtained for minor participants.

### Measures

A self-administered, 10-question survey was developed for the purpose of this study (Table 2). Question 2 (What is your current gender [male, female, nonbinary, other]?) was used to identify participants' gender identity. Question 8 (Do you consider yourself transgender? Yes/No) was used to identify participants who were transgender. Question 5 (Do you perceive things to have additional sensations, such as letters having colors, or sounds having smells? Yes/No) was intended to identify participants who were synesthetes. Question 3 (Do you consider yourself to have a sweet tooth? Yes/No) and Question 9 (Are you color-blind? Yes/No) were included as red herrings to disguise the researcher's hypothesis (i.e., that synesthesia and transgenderism are correlated) from participants.

**Table 2.** **Survey**

Thank you for completing this survey, which may aid in transgender research. EVERYONE is eligible to take this survey, and we seek as many participants as possible. You will remain anonymous—please do not put your name on this paper. If a question is unclear, answer the best you can.
This survey may produce feelings of stress and/or anxiety. Stop the survey if you become uncomfortable.
1. How old are you? ____
If you are under eighteen, please have your parent or guardian put their initials here to show they give permission for you to complete the rest of the survey: ___
2. What is your current gender (male, female, nonbinary, other)? ______________
3. Do you consider yourself to have a sweet tooth? Yes/No
4. Do you take/have you ever taken supplementary testosterone or estrogen? Yes/No
5. Do you perceive things to have additional sensations, such as letters having colors, or sounds having smells? Yes/No
6. Do you believe that you can perceive sensations that other people cannot? Yes/No
7. Are you more sensitive to touch than most people? Yes/No
8. Do you consider yourself transgender? Yes/No
9. Are you color-blind? Yes/No
10. Are you less sensitive to touch than most people? Yes/No

### Data analysis

The survey was distributed to transgender individuals (*n*=96) attending support groups as well as to cisgender participants (*n*=103) identified among individuals accompanying transgender attendees. A chi-square test was performed using a significance level of 0.05.

## Results

**FIG. 1.**
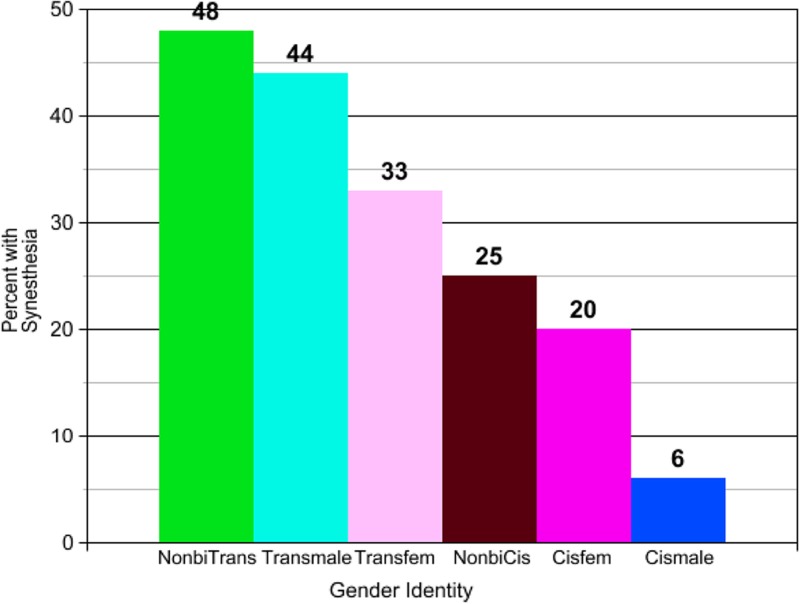
Prevalence of synesthesia by specific gender identity.

A greater number of transgender participants (40/96, 42%) endorsed synesthesia than did cisgender participants (16/103, 16%). The result is significant (*p*=0.000279; Table 3). These findings persisted when analyzed by specific gender identity (i.e., male, female, and nonbinary), with synesthesia most prevalent among nonbinary transgender participants (15/31, 48%) and least prevalent among male cisgender participants (2/33, 6%; Fig. 1). This result is also significant (*p*=0.000852; Table 4).

**Table 3.** **Association of Transgenderism (All Ages) with Synesthesia (*n*=199)**

	Synesthete	Nonsynesthete
Transgender	*n*=41	*n*=55
Transgender binary	25	38
Transgender nonbinary	16	17
Cisgender	*n*=16	*n*=87
Cisgender binary	15	84
Cisgender nonbinary	1	3

The chi-square statistic is 18.9556. The *p*-value is 0.000279. The result is significant at *p*<0.05.

**Table 4.** **Association of Trangenderism (All Gender Identities) with Synesthesia (*n*=199)**

	Synesthete	Nonsynesthete
Transgender	*n*=40	*n*=56
Transgender nonbinary	15	16
Trans male	14	18
Trans female	11	22
Cisgender	*n*=16	*n*=87
Cisfemale	13	53
Cismale	2	31
Cisgender nonbinary	1	3

The chi-square statistic is 20.8823. The *p*-value is 0.000852. The result is significant at *p<0.05.*

**FIG. 2.**
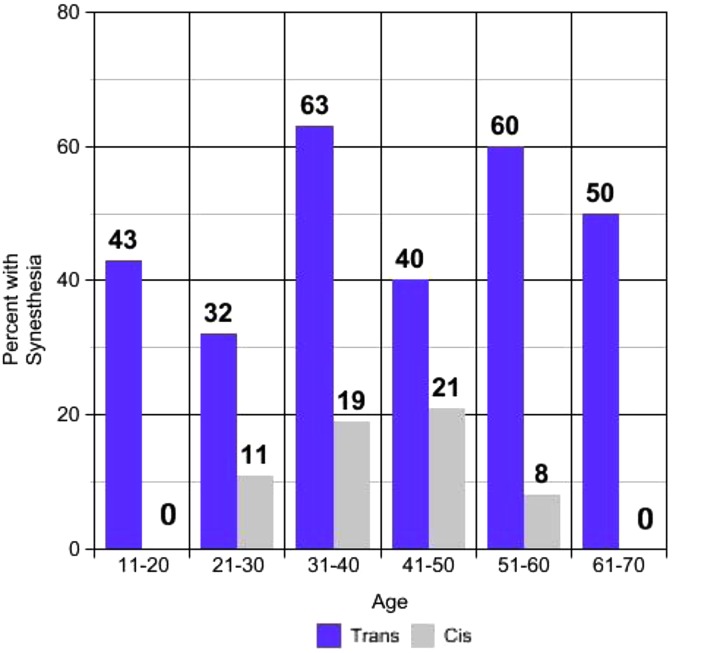
Prevalence of synesthesia by gender identity and age. There were insufficient data (three participants) for ages 8–10 years, for ages 71–90 years (two participants), and for cisgender nonbinary (four participants), so these groups are not included. Two participants did not list their age and also are not included.

Within each decade age group, a higher percentage of transgender participants endorsed synesthesia than did cisgender participants (Fig. 2). Among cisgender participants, synesthesia was most prevalent in decades 31–40 (19%) and 41–50 (21%). *Id*. Among transgender participants, synesthesia was most prevalent in decades 31–40 (63%) and 51–60 (60%). *Id*. For the decade 31–40, the result is significant (*p*=0.013184). Other decades did not produce a significant result.

## Discussion

This study suggests an overall prevalence of synesthesia of 28% (42% in transgender and 16% in cisgender participants). This is a higher prevalence than that found by other studies (i.e., 4.4% in the general population).^3^

This study asked participants, “Do you consider yourself transgender?” Other researchers suggest asking two separate questions (i.e., one for current gender identity and another for birth-assigned gender).^18^

This study asked participants whether they had cross-sensory experiences indicative of synesthesia. Alternatively, participants could be given an objective test for synesthesia.^4^

## Conclusion

This study's results suggest a correlation between transgenderism and synesthesia. The researcher encourages larger scale replication of this study.

Possible practical applications of this area of research include earlier identification of transgender children, which could provide families additional time to support children's gender transitions and make available a greater range of medical treatments to gender-questioning youth.^19^
